# Temporal changes of the respiratory microbiota as cats transition from health to experimental acute and chronic allergic asthma

**DOI:** 10.3389/fvets.2022.983375

**Published:** 2022-08-25

**Authors:** Aida I. Vientós-Plotts, Aaron C. Ericsson, Zachary L. McAdams, Hansjorg Rindt, Carol R. Reinero

**Affiliations:** ^1^College of Veterinary Medicine, University of Missouri, Columbia, MO, United States; ^2^Department of Veterinary Medicine and Surgery, College of Veterinary Medicine, University of Missouri, Columbia, MO, United States; ^3^Comparative Internal Medicine Laboratory, University of Missouri, Columbia, MO, United States; ^4^University of Missouri Metagenomics Center, University of Missouri, Columbia, MO, United States; ^5^Department of Veterinary Pathobiology, College of Veterinary Medicine, University of Missouri, Columbia, MO, United States

**Keywords:** respiratory microbiota, inflammatory airway disease, large animal model, 16S rRNA gene, translational research, gut-lung axis

## Abstract

In humans, deviation from a core airway microbiota may predispose to development, exacerbation, or progression of asthma. We proposed to describe microbiota changes using 16 rRNA sequencing in samples from the upper and lower airways, and rectal swabs of 8 cats after experimental induction of asthma using Bermuda grass allergen, in acute (6 weeks) and chronic (36 weeks) stages. We hypothesized that asthma induction would decrease richness and diversity and alter microbiota composition and structure in the lower airways, without significantly impacting other sites. After asthma induction, richness decreased in rectal (*p* = 0.014) and lower airway (*p* = 0.016) samples. B diversity was significantly different between health and chronic asthma in all sites, and between all time points for lower airways. In healthy lower airways *Pseudomonadaceae* comprised 80.4 ± 1.3% whereas *Sphingobacteriaceae* and *Xanthobacteraceae* predominated (52.4 ± 2.2% and 33.5 ± 2.1%, respectively), and *Pseudomonadaceae* was absent, in 6/8 cats with chronic asthma. This study provides evidence that experimental induction of asthma leads to dysbiosis in the airways and distant sites in both the acute and chronic stages of disease.

This article has been published alongside “Respiratory dysbiosis in cats with spontaneous allergic asthma” (1).

## Introduction

The cat serves as an important animal model for allergic asthma, with both spontaneous and experimental feline asthma, sharing similar features of airway eosinophilia, airway hyperresponsiveness, and airway remodeling as people (2, 3). Asthma is a heterogenous disease in humans, varying by clinical features (phenotype) and inflammatory pathways (endotypes) with the feline model mimicking features of childhood-onset allergic asthma including atopy, T helper 2 lymphocyte driven inflammation with airway eosinophilia, airway hyperresponsiveness and airway remodeling (2, 4). Many environmental influences could tip the balance from a tolerogenic to inflammatory environment and/or sustain airway inflammation. In cats, among the most common allergens involved in asthma development and exacerbation are house dust mites and Bermuda grass, which are also implicated in human allergic asthma (3). Recent studies in humans suggest a role of the respiratory and gut microbiota in the pathogenesis of asthma (5–9).

Historically, the role of bacteria in pulmonary disorders has focused on infection by pathogenic bacteria leading to development, persistence, exacerbation, and/or progression of respiratory diseases (10). However, only 1% of all bacteria can be cultured in the laboratory making culture-independent methods critical for characterizing microbial communities (6). Sequencing bacterial 16S rRNA amplicons, even in low biomass samples such as bronchoalveolar lavage fluid (BALF), represents a powerful tool to overcome a critical barrier to progress in understanding the role of complex microbial communities in the respiratory tract. Recent data suggest the endogenous airway microbiota play a fundamental role in establishing health vs. diseased states with the microbiota having a symbiotic relationship with the host, by providing a barrier function against pathogenic species, regulating cell growth, promoting healing, and modulating immune responses (11, 12). While lower airways of healthy individuals have traditionally been considered sterile because of negative culture results, sequencing of microbial 16S rRNA amplicons has revealed a rich and diverse population of bacterial species previously missed (13, 14). Studies in healthy humans have shown a “core airway microbiota” (5) which differs from asthmatic individuals (6). Similarly, differences in the airway microbiota between healthy and bronchitic dogs have been documented (15). Collectively, these findings suggest that dysbiosis of microbial inhabitants of the respiratory tract occurs with disease and warrants study in cats with asthma.

Chronic glucocorticoid administration is a mainstay in therapy for allergic asthma in humans (16) and in cats (17), yet it does not reverse the underlying immunopathology of allergic asthma and can be associated with many side effects. Understanding differences in microbial communities in healthy vs. inflammatory airways represents the first step in trying to determine if respiratory dysbiosis exists. Furthermore, in patients with allergic asthma, it sets the stage for future studies to determine if the microbiota can be modulated to restore immunologic tolerance and attenuate disease. This study aimed to characterize the richness and composition of the upper and lower airway microbiota and the gastrointestinal microbiota in cats as they transitioned from health to experimentally induced acute and chronic asthma. Using an experimental model of allergic asthma, we hypothesized that as in people, there would be a significant decrease in microbial richness and diversity, as well as alterations in microbial community composition and structure of the lower airways as cats transitioned from health to acute and chronic asthma, without significantly impacting the microbiota of the upper airways or gastrointestinal tract.

## Materials and methods

### Ethics statement

All studies were performed in accordance with the Guide for the Use and Care of Laboratory Animals and were approved by the University of Missouri Institutional Animal Care and Use Committee (MU IACUC protocol #7891).

### Cats

Cats were bred from a colony (Comparative Internal Medicine Laboratory, University of Missouri, Columbia, MO). Eight 3-year old cats (4 males and 4 females) were included. Cats were housed together in large runs with elevated platforms for climbing and enrichment toys. Access to food and clean drinking water were provided *ad libitum*. To reduce risk of aspiration, cats were fasted for a minimum of 12 h prior to anesthesia for sample collection.

Cats were determined to be healthy by absence of respiratory clinical signs, a normal physical examination by a board-certified veterinary internal medicine specialist and lack of cytologic evidence of infection or inflammation from bronchoalveolar lavage fluid (BALF) samples. Cats did not receive any medications (steroids or bronchodilators) during the study period, nor did they require medications long term. Euthanasia was not an endpoint of the study; all cats were subsequently adopted into private homes.

### Sample collection

Rectal, oropharyngeal (OP) swabs, and BALF were collected at the beginning of the study (baseline; health), and after 6 (acute asthma) and 36 (chronic asthma) weeks of experimental asthma induction. Asthma induction was achieved using Bermuda grass allergen (BGA) *via* subcutaneous injection with alum, intranasal administration, and delivery of aerosols, as previously described (2). Confirmation of an asthmatic phenotype was made *via* positive intradermal test to BGA and airway eosinophilia (≥16% eosinophils in BALF). Samples were collected according to previously published protocols (14). Briefly, cats were anesthetized, and prior to intubation, a sterile swab was used to rub the caudodorsal aspect of the oropharynx, while avoiding contact with the rest of the oral cavity. Cats were then carefully intubated using a sterile 3.5–4 French endotracheal tube. To collect BALF, a 20 mL aliquot of sterile saline was instilled and aspirated *via* a sterile 8 French red rubber catheter that was threaded through the endotracheal tube until it was gently wedged in an airway. Once anesthetized, while avoiding contact with the perianal area, a sterile cotton swab was inserted at a minimum of 4 cm rectally to obtain a sample. Sterile saline and swabs were included as controls. Immediately after collection, all samples were placed on ice and transported to the laboratory. Promptly after collection, samples were centrifuged to pellet bacterial cells. Supernatant was discarded and pellets were resuspended in 800 μL lysis buffer adapted from Yu et al. (4% sodium dodecyl sulfate, 50 mM EDTA, 500 mM NaCl, and 50 mM Tris-HCl pH 8.0) (18). All samples were banked at −80°C until the end of the study, and DNA was extracted as a single batch.

### DNA extraction, 16S rRNA library preparation, sequencing, and informatics

DNA from rectal, OP and BALF was extracted using the column method as previously described (14, 19). Library construction and sequencing was completed at the University of Missouri DNA Core facility as previously described (19). Assembly, filtering, binning, and annotation of DNA sequences were performed at the MU Informatics Core using Quantitative Insights Into Microbial Ecology 2 (QIIME 2) v2021.2 (20). Briefly, sequences were trimmed of the Illumina adapters with cutadapt (21). Using DADA2 (22), trimmed forward and reverse reads were truncated to 150 base pairs, paired, then denoised into unique sequences called Amplicon Sequence Variants (ASVs). A feature table containing the frequency of each ASV per sample was rarefied to 1,488 total features per sample maximizing the number of subsampled features per sample and total number of samples retained for further analysis. Rarefaction cutoffs are determined as one less than the lowest read count for any given sample >1,000 reads. Here, the lowest read count >1,000 was 1,489 reads, therefore subsampling was performed at one less than that and samples with a total feature number <1,488 were omitted from downstream analyses. Taxonomy was assigned to each unique ASV with a sklearn algorithm (23) using the QIIME2-provided 99% non-redundant SILVA v132 reference database of the 515F/806R region of the 16S rRNA gene (20, 24).

### Statistical analysis

Statistical analysis was performed using Sigma Plot 14.0 (Systat Software Inc., Carlsbad, CA) and R v4.1.2 (25). BALF eosinophil counts in healthy and asthmatic cats were compared *via t*-test. For coverage and richness of rarefied sequences, normality was first tested using the Shapiro-Wilk method and equal variance was tested using the Brown-Forsyth method. Alpha-diversity indices were determined using the vegan v2.5.7 (26) package within R v4.1.2 (25). Repeated measures ANOVA on ranks was used to test for differences between sample sites in coverage, richness, α diversity, and relative abundance of all taxa at the level of family from any one sample site using Sigma Plot 14.0. *Post-hoc* comparisons were made *via* Tukey's method. Differences in β-diversity between time-points were tested using PERMANOVA and principal coordinate analysis (PCoA) of a quarter-root transformed feature table using Bray-Curtis similarities was performed using the vegan (v2.5.7) (26) package within R v4.1.2 (25). Differential abundance testing was performed between healthy and chronic groups using a family feature table pseudocounted by one using ALDEx2 (27) and analysis of composition of microbiomes (ANCOM) (28) using default parameters within R v4.1.2 (25). Differentially abundant families were identified by ANCOM as those with a W score in the 90th percentile or greater. Results were presented as mean ± SEM, and considered statistically significant for *p*-values ≤ 0.05. For time points within each site that significantly differed from health, a volcano plot was generated using EnhancedVolcano (29) plotting the Benjamini-Hochberg corrected *p*-value from the Welch's *t* test performed within ALDEx2. Microbiome analyses can be found at https://github.com/ericsson-lab/experimental_asthma.

## Results

### Induction of acute and chronic experimental allergic asthma was associated with airway eosinophilia

BALF eosinophil counts in healthy cats ranged from 2.5 to 6.5% (mean ± SEM; 4.6 ± 0.5%). After asthma induction there was a significant increase in airway eosinophilia in both the acute and chronic stages of disease compared to health (60.1 ± 4.5% and 51.6 ± 6.7%, respectively; *p* < 0.001).

### Depth of microbiota coverage was unchanged after asthma induction and was lowest in BALF samples

First, we compared the number of high-quality sequence reads (i.e., coverage) obtained for each group and time point. Over the course of the study, rectal and OP samples had significantly greater depth of sequencing coverage compared to BALF (mean ± SEM of 178,463 ± 25,059 and 77,855 ± 9,823 and 4,558 ± 519 sequences/sample, respectively *p* < 0.001). Although BALF with its lower biomass had significantly lower sequencing coverage than OP and rectal samples (*p* < 0.0001), alpha rarefaction curves indicated ample feature coverage (Supplementary Figure 1A). Good's coverage of the rarefied feature table estimated >97% community sampling across all sites and time points (Supplementary Figure 1B). Regarding the negative controls, sterile saline and swabs, yielded significantly less reads per sample 1,986 ± 233 (*p* = 0.003) than BALF samples, and PERMANOVA of Bray Curtis similarity index showed that they were significantly different than any other site.

### Richness significantly decreased in rectal and lower airway samples in chronic asthma

**Figure 1:**
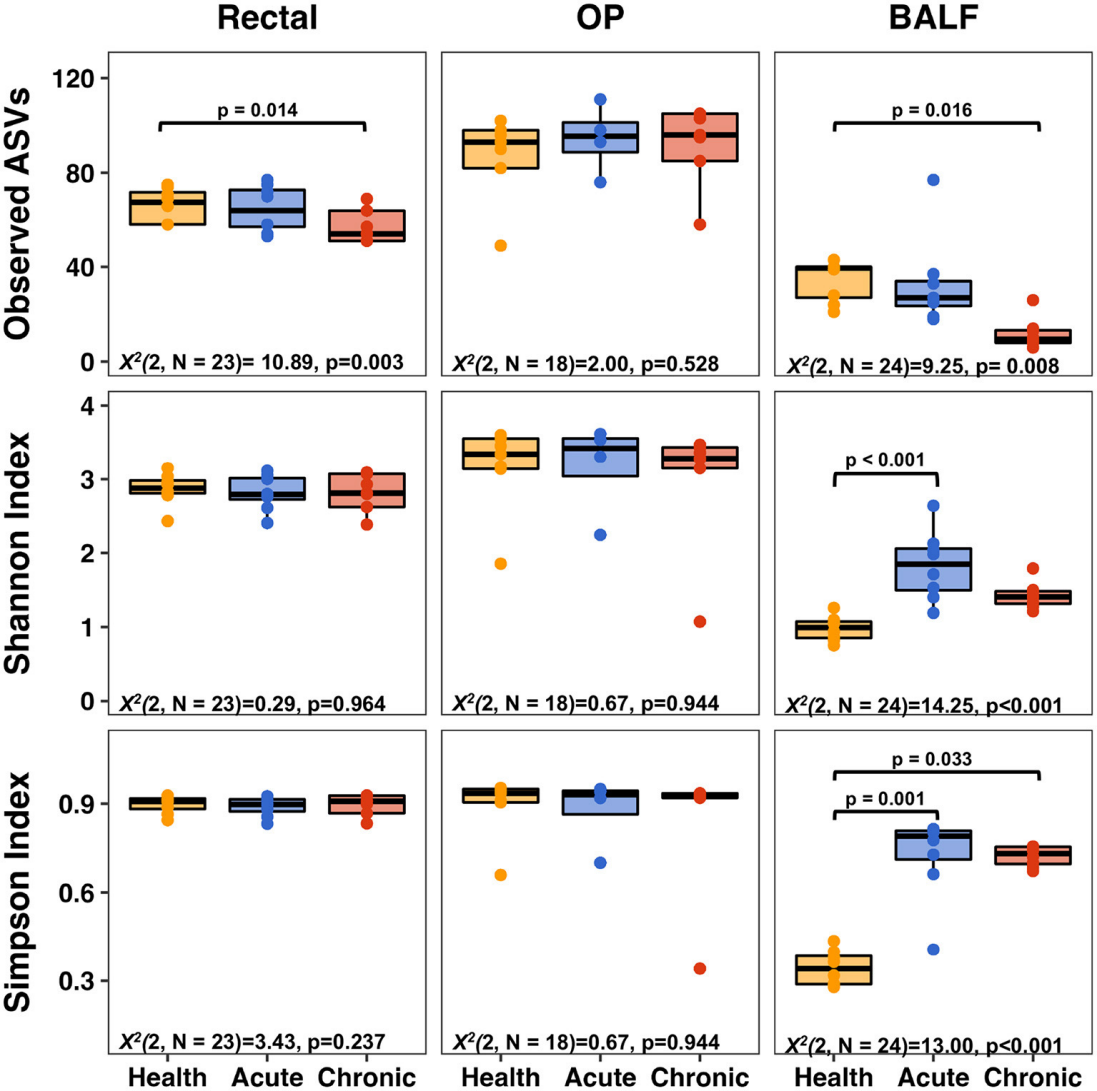
Alpha diversity metrics—richness (top row), Shannon Index (middle row), and Simpson Index (bottom row) of rectal (left column), upper airways (OP, middle column) and lower airways (BALF, right column). Significant decreases in richness were observed in BALF and rectal samples when comparing health to chronic asthma. Within sample diversity in BALF was significantly increased in acute and chronic asthma. OP, oropharyngeal; BALF, bronchoalveolar lavage fluid.

While no difference in richness was detected between health and acute asthma, rectal samples from chronic asthma revealed significantly decreased richness in observed ASVs from healthy cats (from 66 ± 7 to 57 ± 7, respectively; *p* = 0.014). In the upper airways, there was no significant difference in richness between health and acute or chronic asthma (*p* = 0.528). In contrast, in the lower airways there was no significant change from health to acute asthma (34 ± 8, 33 ± 17; *p* = 1.0) however, there was a significant decrease in richness from health (34 ± 8) to chronic asthma (12 ± 6; *p* = 0.016). Within sample diversity significantly increased in the lower airways in the acute (Simpson Index: *p* < 0.001; Shannon Index: *p* < 0.001) and chronic stages of disease (Simpson Index: *p* = 0.033), but it was unchanged at other sites (Figure 1).

### Microbial community structure at each site significantly differed from other sites, and was significantly altered for all sites in chronic asthma compared to health

**Figure 2:**
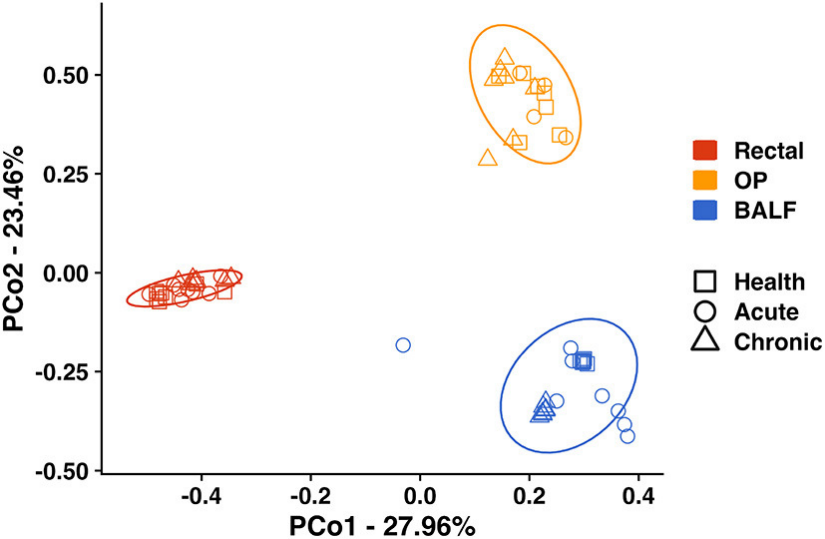
Principal coordinate analysis including all sites—Principal coordinate analysis of Bray Curtis similarity index including all sites (Rectal—red; OP—orange; BALF—blue) in health (squares), in acute asthma (6 weeks) (circles) and chronic asthma (36 weeks) (triangles) after experimental asthma induction showed significant differences in microbial community structure between sites. The ellipses represent a 95% confidence interval. OP, oropharyngeal; BALF, bronchoalveolar lavage fluid.

**Figure 3:**
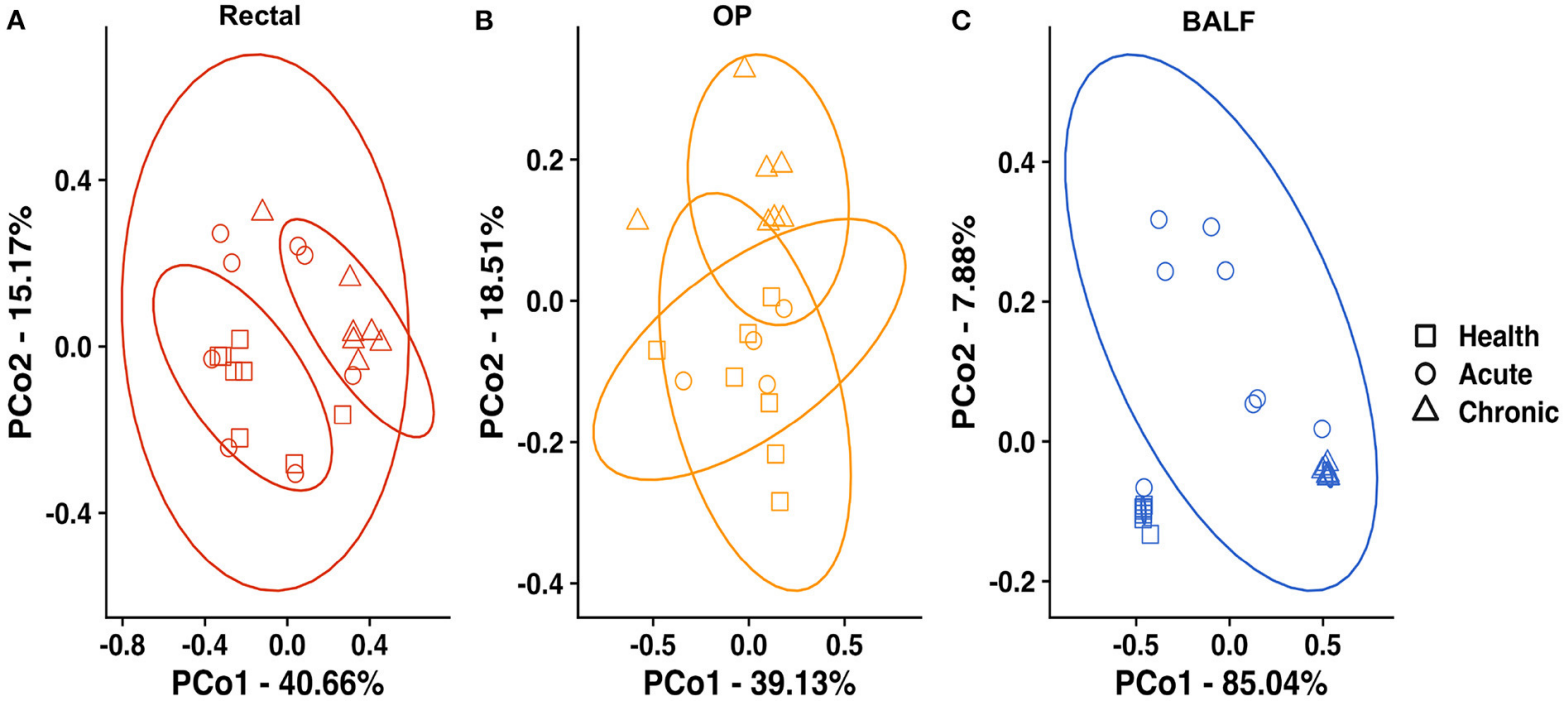
Principal coordinate analysis at each site—of Bray Curtis similarity index in rectal samples **(A)**, OP swabs **(B)** and BALF **(C)** in healthy cats at baseline (squares), acute asthma (6 weeks) (circles) and chronic asthma (36 weeks) (triangles). PERMANOVA showed a significant difference in community composition at all sites between health and chronic asthma, as well as health and acute asthma in the lower airways. Ellipses represent 95% confidence interval. OP, oropharyngeal; BALF, bronchoalveolar lavage fluid.

Principal coordinate analysis (PCoA) was used to assess β-diversity of microbial communities found at all sample sites collectively. When samples from all sites at baseline and after induction of acute and chronic asthma were included in the analysis, the samples clustered by site (Figure 2). Testing for main effects *via* two-way PERMANOVA found significant main effects of both sample site (*p* = 0.0001; F = 49.65) and time point (*p* = 0.0001; F = 17.08), as well as a significant interaction between site and time point (*p* = 0.0001; F = 11.85). Recognizing that the inherent and well-known structural differences between microbial communities present in the sites tested could obscure time-dependent differences within each sample site, PCoA of samples from each site were independently performed. For rectal samples, PCoA plots showed greater variation and overlap between acute asthma vs. the other two time points, however there was minimal overlap between health and chronic asthma (Figure 3A). This observation was supported by PERMANOVA analysis which showed there was a significant difference in structure of the rectal samples between health and chronic asthma (*p* = 0.0003; F = 5.88). While there was overlap between time points within the upper airways, OP samples clustered more tightly (Figure 3B), and PERMANOVA showed there was a significant difference between health and chronic asthma (*p* = 0.011; F = 2.93). In contrast to the other two sites, there was no overlap between BALF samples from health compared to either acute or chronic asthma (Figure 3C). Pairwise comparisons revealed significant differences in microbial structure in the lower airways between health and acute (*p* = 0.0004) and health and chronic asthma (*p* = 0.0004).

### Asthma induction impacted the relative abundance of predominant taxa especially in the lower airways

At the level of family, a total of 116 taxa were sequenced in at least one rectal sample at any time point. Of these, 24/116 were present at an overall relative abundance of at least 0.5% and 17/24 accounted for >90% of the microbial community (Table 1). In healthy cats at baseline, the most abundant families in rectal samples were *Lachnospiraceae and Veillonellaceae* (phylum Firmicutes), *Bacteroidaceae* (phylum Bacteroidetes), *Helicobacteraceae* (phylum Epsilonbacteraeota) and *Fusobacteriaceae* (phylum Fusobacterium). The most significant changes in relative abundance were observed in chronic asthma with a significant decrease in Firmicutes from 47.1 ± 1.4% to 25.2 ± 1.6% (*p* = 0.033) and an increase in Proteobacteria from 5.8 ± 0.5% to 12.5 ± 1.2% (*p* = 0.003). At the level of family, a total of 27 taxa at were sequenced in at least 1 OP sample at any time point. Of these, 22/27 were present at an overall relative abundance of at least 0.5% and 16/27 accounted for >90% of the microbial community (Table 2). *Pasteurellaceae* (phylum Proteobacteria) and *Porphyromonadaceae* (phylum Bacteroidetes) were the most abundant taxa in the upper airways. There was no significant change in relative abundance of the aforementioned taxa over time (*p* > 0.05), with the exception of *Pseudomonadaceae*, which significantly decreased from health to chronic asthma (*p* = 0.008).

**Table 1:** Relative abundance (mean ± SEM%) of taxa at the level of family present in any rectal sample at any time point at >0.5% accounting for >90% of the overall microbial community.

**Phylum**	**Family**	**Health (day 0)**	**Acute asthma (week 6)**	**Chronic asthma (week 36)**	***p*** **-value**
**Rectal samples**					
Actinobacteria		7.0 ± 0.4	8.3 ± 0.7	8.6 ± 0.9	0.53
	*Bifidobacteriaceae*	2.9 ± 1.2	2.8 ± 0.9	0.6 ± 0.3	0.06
	*Atopobiaceae*	0.2 ± 0.05	**4.5 ± 1.3**	0.8 ± 0.4	**0.003**
Bacteroidetes		18.1 ± 0.1	19.4 ± 1.1	24.5 ± 1.7	0.61
	*Bacteroidaceae*	10.4 ± 1.0	13.2 ± 3.3	21.2 ± 2.8	0.13
	*Porphyromonadaceae*	2.4 ± 2.0	2.4 ± 1.5	**6.0 ± 2.2**	**0.03**
	*Prevotellaceae*	4.9 ± 2.1	2.8 ± 1.3	0.6 ± 0.3	0.08
Epsilonbacteraeota		11.4 ± 0.8	12.0 ± 1.4	4.9 ± 1.4	0.53
	*Campylobacteraceae*	3.7 ± 1.1	7.1 ± 2.2	5.6 ± 4.0	0.79
	*Helicobacteraceae*	7.7 ± 2.2	4.1 ± 2.0	**0.01 ± 0.01**	**0.008**
Firmicutes		47.1 ± 1.4	41.0 ± 1.7	**25.2 ± 1.6**	**0.033**
	*Lachnospiraceae*	14.4 ± 3.3	20.3 ± 5.8	5.6 ± 3.0	0.12
	*Veillonellaceae*	20.2 ± 3.2	11.4 ± 3.6	**6.3 ± 1.3**	**0.033**
	Class *Clostridiales—*Family XI	1.4 ± 0.9	4.6 ± 2.5	**11.7 ± 3.1**	**0.016**
	*Erysipelotrichaceae*	4.3 ± 1.7	1.2 ± 0.4	**0.6 ± 0.2**	**0.016**
	*Ruminococcaceae*	2.1 ± 0.9	1.6 ± 0.6	0.9 ± 0.3	0.355
	*Acidaminococcaceae*	3.7 ± 0.5	1.4 ± 0.5	**0.3 ± 0.2**	**<0.001**
Fusobacteria		10.5 ± 1.0	8.7 ± 0.8	11.7 ± 0.8	0.79
	*Fusobacteriaceae*	10.5 ± 3.0	8.0 ± 2.3	13.4 ± 1.5	0.79
Proteobacteria		5.8 ± 0.5	10.7 ± 0.6	**12.5 ± 1.2**	**0.003**
	*Pasteurellaceae*	0.2 ± 0.1	1.6 ± 1.0	**6.9 ± 2.6**	**0.016**
	*Burkholderiaceae*	1.7 ± 0.3	1.8 ± 0.5	3.6 ± 0.7	0.12
	*Enterobacteriaceae*	1.3 ± 1.0	1.3 ± 0.8	2.9 ± 2.2	0.07

Bold text indicates a significant change (*p* < 0.05) in relative abundance compared to baseline.

**Table 2:** Relative abundance (mean ± SEM) of taxa at the level of family present in any upper airway sample at any time point at >0.5% accounting for >90% of the overall microbial community.

**Phylum**	**Family**	**Health (day 0)**	**Acute asthma (week 6)**	**Chronic asthma (week 36)**	***p*** **-value**
**OP samples**					
Bacteroidetes		29.5 ± 1.0	21.5 ± 1.7	23.9 ± 1.8	0.49
	*Bacteroidaceae*	3.6 ± 0.6	2.6 ± 0.8	2.0 ± 0.4	0.15
	*Chitinophagaceae*	1.0 ± 0.4	2.7 ± 1.9	5.1 ± 2.7	0.39
	*Flavobacteriaceae*	4.9 ± 1.5	2.1 ± 0.6	2.7 ± 0.6	0.33
	*Porphyromonadaceae*	13.0 ± 1.9	11.2 ± 3.1	10.1 ± 1.7	0.41
	*Prevotellaceae*	2.8 ± 0.5	2.1 ± 0.5	3.1 ± 0.8	0.69
	*Weeksellaceae*	3.5 ± 0.5	2.0 ± 0.8	3.9 ± 0.7	0.79
Firmicutes		6.6 ± 0.2	6.1 ± 0.6	6.6 ± 0.5	0.82
	*Erysipelotrichaceae*	1.2 ± 0.2	1.1 ± 0.4	2.3 ± 0.5	1.00
	*Lachnospiraceae*	1.1 ± 0.2	1.1 ± 0.3	1.1 ± 0.2	0.15
	*Peptostreptococcaceae*	0.8 ± 0.2	1.9 ± 0.6	0.7 ± 0.1	0.15
Fusobacteria		5.7 ± 0.6	3.3 ± 0.4	2.2 ± 0.2	0.25
	*Fusobacteriaceae*	4.1 ± 1.4	9.4 ± 6.5	1.1 ± 0.2	0.12
	*Leptotrichiaceae*	1.7 ± 0.4	0.8 ± 0.2	1.5 ± 0.4	0.97
Proteobacteria		55.4 ± 0.9	52.7 ± 3.7	49.9 ± 3.4	0.35
	*Burkholderiaceae*	0.9 ± 0.1	1 ± 0.2	1.4 ± 0.3	0.97
	*Cardiobacteriaceae*	1.0 ± 0.4	1.1 ± 0.5	2.5 ± 0.9	0.53
	*Moraxellaceae*	9.3 ± 1.5	6.8 ± 1.4	8.2 ± 2.0	0.79
	*Neisseriaceae*	7.6 ± 1.2	5.9 ± 1.6	10.2 ± 2.7	0.90
	*Pasteurellaceae*	27.8 ± 5.1	20.8 ± 6.7	32.5 ± 8.4	0.10
	*Pseudomonadaceae*	6.5 ± 1.0	12.8 ± 5.1	**0.1 ± 0.04**	**0.008**

Bold text indicates a significant change (*p* < 0.05) in relative abundance compared to baseline. OP, oropharyngeal.

**Figure 4:**
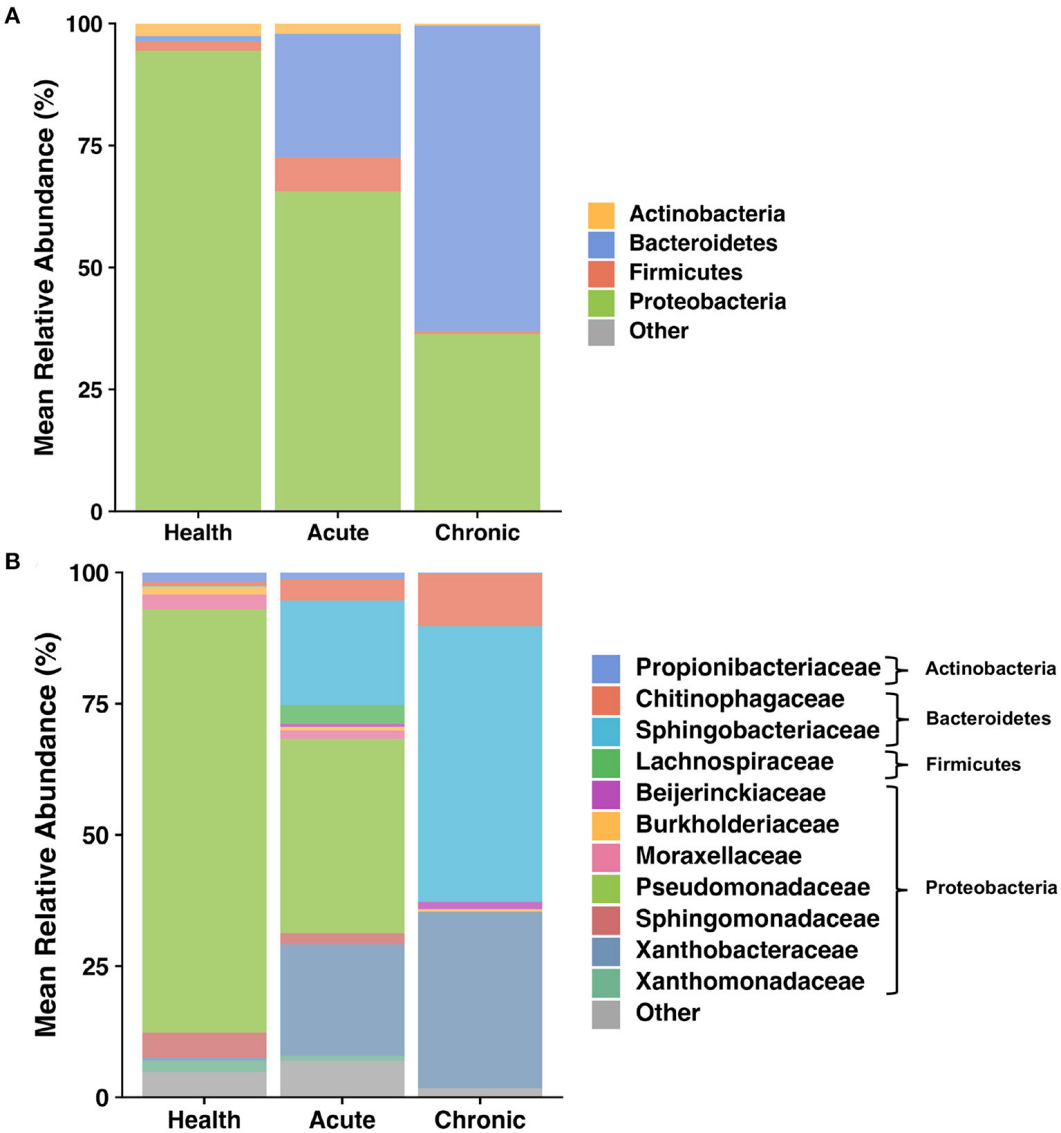
Taxa present in BALF—Mean relative abundance of taxa present at >1% in bronchoalveolar lavage fluid (BALF) collected as 8 cats transitioned from health to acute and chronic experimental asthma, annotated to the taxonomic level of phylum **(A)** and family **(B)**.

**Figure 5:**
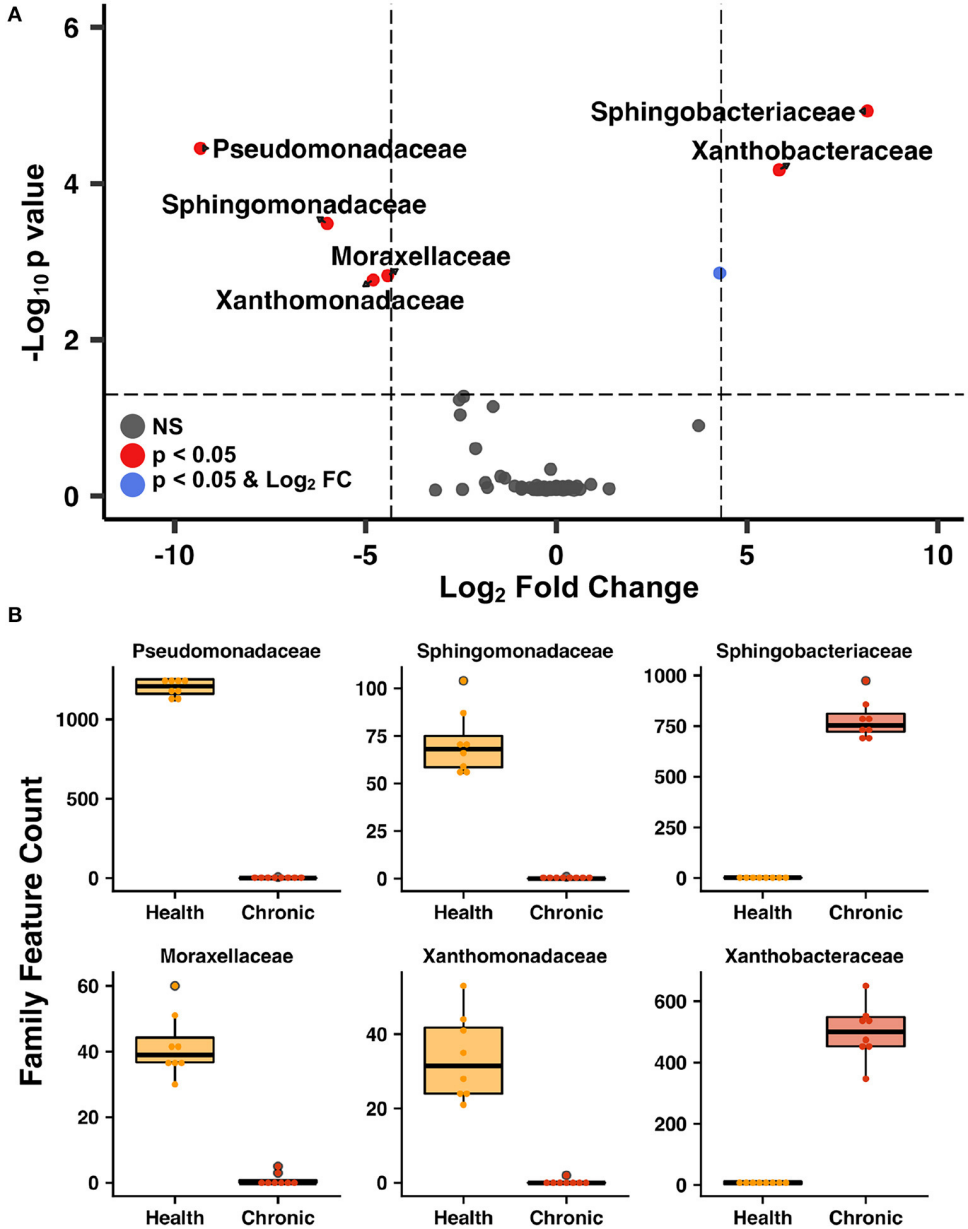
Lower airway taxa that underwent most significant changes after asthma induction—Volcano plot **(A)** highlighting the most abundant families in the lower airways that underwent significant changes (*p* < 0.05) and a 20-fold change in abundance as cats transitioned from health to chronic asthma. Box plots of bacterial taxa present at >0.5% relative abundance **(B)** that underwent at least a 20-fold change as the lower airways transitioned from health to chronic asthma.

The lower airways underwent the greatest changes in community composition after experimental asthma induction. In health, the feline lower airways were primarily composed of taxa from the phylum Proteobacteria (94.4 ± 0.3%) which significantly decreased to 62.2 ± 1.9% and 36.1 ± 0.8% in acute and chronic asthma, respectively (*p* < 0.001). Conversely, taxa from the phylum Bacteroidetes, significantly increased from 1.3 ± 0.3% in health to 25.5 ± 2.1% and 62.9 ± 1.0% in acute and chronic asthma, respectively (*p* = 0.003; *p* < 0.001) (Figure 4A). At the family level, 63 total taxa at were sequenced in at least 1 sample of the lower airways at any time point. Of these, 12/63 were present at an overall relative abundance of at least 0.5%, and 6/12 accounted for >90% of the microbial community at any time point (Table 3). The relative abundance of *Pseudomonadaceae, Sphingobacteriaceae, Moraxellaceae*, and *Xanthobacteraceae* underwent significant changes as cats transitioned from health to acute and chronic asthma. In health, the lower airway microbiota was dominated by *Pseudomonadaceae* and while this was still the predominant taxa in acute asthma, this time point was characterized by a decrease in relative abundance of this taxon and an increase in *Sphingobacteriaceae* and *Xanthobacteraceae*. In chronic asthma, *Sphingobacteriaceae* and *Xanthobacteraceae* predominated, while *Pseudomonadaceae* was not identified in the lower airway of 6/8 cats (Figure 4A and Supplementary Figure 2). A volcano plot was used to visually identify and generate box plots of taxa that underwent significant changes (*p* < 0.05) and had >20-fold change in relative abundance (Figures 5A,B). Differentially abundant taxa identified with ALDEx2 in health or chronic asthma were corroborated as differentially abundant with ANCOM (see Supplementary Table 1).

**Table 3:** Relative abundance (mean ± SEM) of taxa at the level of family and genus present in any lower airway sample at any time point at >0.5% accounting for >90% of the overall microbial community.

**Phylum**	**Family**	**Health (day 0)**	**Acute asthma (week 6)**	**Chronic asthma (week 36)**	***p*** **-value**
	**Genus**				
**BALF samples**					
Proteobacteria		94.4 ± 0.3	**65.2 ± 1.9**	**36.1 ± 0.8**	**<0.001; <0.001**
	*Pseudomonadaceae*	80.4 ± 1.3	36.7 ± 7.1	**0.1 ± 0.1**	**<0.001**
	*Pseudomonas*	80.6 ± 1.3	37.0 ± 7.3	**0.1** ±**0.1**	**<0.001**
	*Xanthobacteraceae*	0.6 ± 0.1	21.5 ± 5.1	**33.5 ± 2.1**	**0.008**
	*Rhodopseudomonas*	0.0 ± 0.0	12.5 ± 4.4	**33.6** ±**2.1**	**<0.001**
	*Chitinophagaceae*	0.8 ± 0.8	4.2 ± 1.8	10.2 ± 3.5	0.075
	*Filobacterium*	0.7 ± 0.7	1.3 ± 1.3	**9.9 ± 3.4**	**0.02**
	*Sphingomonadaceae*	4.7 ± 0.3	2.0 ± 0.3	**0.0 ± 0.0**	**<0.001**
	*Sphingobium*	4.4 ± 0.4	2.0 ± 0.3	**0.0 ± 0.0**	**<0.001**
	*Moraxellaceae*	3.0 ± 0.2	1.6 ± 0.2	**0.1 ± 0.0**	**<0.001**
	*Acinetobacter*	2.8 ± 0.2	1.5 ± 0.3	**0.0** ±**0.0**	**<0.001**
	*Xanthomonadaceae*	2.4 ± 0.2	0.9 ± 0.1	**0.0 ± 0.0**	**<0.001**
	*Stenotrophomonas*	2.3 ± 0.3	0.9 ± 0.1	**0.0** ±**0.0**	**<0.001**
	*Burkholderiaceae*	1.4 ± 0.1	0.7 ± 0.2	**0.4 ± 0.3**	**0.017**
	*Delftia*	1.4 ± 0.1	0.5 ± 0.1	**0.0** ±**0.0**	**0.002**
	*Beijerinckiaceae*	0.0 ± 0.0	0.5 ± 0.2	**1.4 ± 0.2**	**0.008**
	*Methylobacterium*	0.0 ± 0.0	0.6 ± 0.2	**1.2 ± 0.2**	**0.004**
Bacteroidetes		1.3 ± 0.3	**25.5 ± 2.1**	**62.9 ± 1.0**	**0.003; <0.001**
	*Sphingobacteriaceae*	0.1 ± 0.0	19.7 ± 7.2	**52.4 ± 2.2**	**0.002**
	*Mucilaginibacter*	0.0 ± 0.0	10.2 ± 3.8	**28.6 ± 2.2**	**<0.001**
	*Mucilaginibacter ginsengisoli*	0.0 0.0	9.6 3.5	**23.9 0.7**	**<0.001**
Firmicutes		1.9 ± 0.2	7.3 ± 1.5	0.7 ± 0.2	0.063
	*Lachnospiraceae*	0.0 ± 0.0	3.7 ± 3.6	0.0 ± 0.0	0.120
	*Roseburia*	0.0 ± 0.0	2.3± 2.2	0.0 ± 0.0	0.18
	*Lachnoclostridium*	0.0 ± 0.0	0.7 ± 0.7	0.0 ± 0.0	0.22
	*Streptococcaceae*	0.5 ± 0.1	0.8 ± 0.2	0.5 ± 0.4	0.05
	*Streptococcus*	0.5 ± 0.1	0.1 ± 0.1	0.5 ± 0.5	0.06
	*Streptococcus salivarius subsp. thermophilus*	0.0 ± 0.0	0.7 ± 0.2	0.0 ± 0.0	0.125
Actinobacteria		2.5 ± 0.1	2.1 ± 0.2	**0.4 ± 0.1**	**0.005**
	*Propionibacteriaceae*	1.8 ± 0.3	1.2 ± 0.5	**0.3 ± 0.2**	**0.008**
	*Cutibacterium*	1.8 ± 0.3	1.3 ± 0.5	**0.3 ± 0.2**	**0.008**

Bold text indicates a significant change (*p* < 0.05) in relative abundance compared to baseline. BALF, bronchoalveolar lavage fluid.

## Discussion

Use of an experimental model of acute and chronic feline allergic asthma provides a unique opportunity to document temporal changes of the respiratory and gastrointestinal microbiota as cats transition from health to asthma. Allergic asthma is orchestrated by allergen-specific T helper 2 lymphocytes which, in genetically susceptible individuals with certain environmental influences, trigger and amplify a local inflammatory response to what should be benign aeroallergens (3, 30). As it is not feasible to repetitively and invasively sample human airways prior to and after development of asthma, utilizing this large animal model as a surrogate can help elucidate key changes in the respiratory and gastrointestinal microbiota critical to an understanding of host-resident microbe interactions in allergic airway disease. In the current study, as cats transitioned from health to chronic asthma, there was a significant decrease in microbial richness in both the lower airways and the gastrointestinal tract and increased α diversity in the lower airways. Using PCoA, microbial community composition was found to be unique at each site (upper airways, lower airways, and the rectum) and clustered by site from health to induction of asthma. However, after asthma induction, the most profound compositional changes were noted in the lower airways, the primary site of inflammation in this model. These changes were exemplified by alterations in the relative abundance of predominant taxa found in healthy cats at baseline (14).

Animals including mice, rats, guinea pigs, rabbits, dogs, cats, sheep, horses, and non-human primates have been used as induced or spontaneous models of asthma (2, 31, 32). Mice are used most frequently likely due to ease of genetic manipulations, large immunology toolkit, simple handling, rapid reproduction rate, availability, and expense. However, there are documented differences in inflammatory response, lung function, and lung histology (33, 34) between mice and humans. Importantly, clinical treatments showing promise in rodent models have often had poor translational success in human clinical trials (35). While induced murine models of asthma have provided valuable data that has helped further the understanding of the mechanisms of disease and identify potential therapeutic intervention, the feline model of allergic asthma stands out as a better translational model for several reasons. First, the cat spontaneously develops allergic asthma with hallmark clinical, immunologic, and pathologic features as humans (2). Cats share the same environmental exposures as humans and respond to aeroallergens with a similar pathogenesis (3). Spontaneous feline asthma is most like childhood onset asthma, and experimentally induced feline asthma mimics both conditions (3). Second, cats have more anatomic similarities to humans relevant to study of asthma (3, 36). Third, longitudinal studies are facilitated in cats because of their longer lifespan and their bigger size; compared to mice, a bigger size facilitates instrumentation (e.g., for direct pulmonary mechanics measures), collection of larger quantities of samples (e.g., blood, BALF), and allows serial collection of invasive samples like BALF and direct pulmonary mechanics in the same animal without requirement for terminal experiments (2). Fourth, tachyphylaxis or tolerance to allergen with chronic exposure occurs in mice (37) but not cats (2) making chronic allergen-induced models of asthma challenging to study in rodents. Preclinical trials of experimental therapies such as tyrosine kinase inhibitors, adipose-derived mesenchymal stem cells, and allergen-specific immunotherapy in the feline allergic asthma model have clinical relevance to humans with respect to therapeutic efficacy (38–42). While there are no published studies on the respiratory microbiota in experimental or spontaneous feline asthma, healthy cats have site-specific microbial communities in the upper and lower respiratory tract, gastrointestinal tract, and feces (14) and further, administration of an oral probiotic significantly changed the microbial community composition in the lower airways of healthy cats (43). These studies set the stage for investigation of respiratory and gastrointestinal dysbiosis (imbalance in microbial communities associated with disease), and ultimately, manipulation of the microbiota at either site as a novel therapeutic strategy.

Development of asthma is linked to genetic and environmental contributors, with the latter helping explain dramatic increases in asthma prevalence in developed countries over the past few decades (44). In childhood onset asthma, allergic sensitization is influenced by early-life events that can cause gut dysbiosis including antibiotic use, delivery by cesarean-section, formula feeding instead of breast feeding, and urban vs. farm living (45). Thus, early life alterations in the gut microbiota have been postulated to negatively impact immune development increasing risk for asthma development in children. When followed over time, infants with lower abundances of key bacterial taxa reflective of gut dysbiosis had a high risk of asthma diagnosis by 3 years of age (45). Production of bacterial metabolites in the gut that can promote specific immune responses at distant sites (e.g., lungs) may help explain communication of the gut-lung axis (46). In the current study prior to induction of asthma when cats had documented absence of airway inflammation, microbial composition of the rectal samples observed at the level of phylum was similar to a previous study in healthy cats and in healthy humans with Firmicutes and Bacteroidetes predominating (14, 47). When comparing the feline gut microbiota in health vs. chronic asthma, there was a significant decrease in Firmicutes and increase in Proteobacteria. Similar changes in the intestinal microbiota after experimental allergic airway disease induction have also been reported in mice (48). Another study in a murine asthma model investigated the airway microbiome in interleukin 13-rich lung environment and alterations to the gut microbiome; results supported chronic airway inflammation induced by IL-13 can cause both airway and gastrointestinal dysbiosis (49). Collectively, these studies support the bidirectional nature of the interactions between the intestinal and respiratory microbiota and highlights that microbial populations at individual sites in the body are not independent of each other.

Respiratory dysbiosis, a deviation from the microbiota in health, is well-documented in humans with asthma (6, 50), as well as cystic fibrosis (51, 52), COPD, and other respiratory diseases (53, 54). In healthy cats at baseline, the lower airways were mostly composed of Proteobacteria, primarily from the family *Pseudomonadaceae*. This contrasts with several studies in healthy people showing a higher relative abundance of Bacteroidetes and Firmicutes in the airways (55–58). Taking into consideration all the factors that contribute to the development of the healthy microbiota such as repetitive microaspiration, the upper airway microbiota, environment, diet, and genetics (57), it is not surprising that the lower airway microbiota composition is different between the two species. Respiratory dysbiosis in cats with acute and chronic asthma was characterized by a significant change in relative abundance of bacterial taxa (Table 3, Figure 4), shifting from 94.4% Proteobacteria and 1.3% Bacteroidetes in health to 65.2% and 36.1% Proteobacteria and 25.5% and 62.9% Bacteroidetes in acute and chronic asthma, respectively. Furthermore, in chronic feline asthma four families present in > 0.5% relative abundance in health had ≥20-fold changes (Figure 5B). Compared to health, in chronic feline asthma, relative abundance within the phylum Proteobacteria changed with the family *Xanthobacteraceae* increasing but the families *Pseudomonadaceae* and *Moraxellaceae* decreasing. Direct comparison of the feline allergic asthma model to human asthmatic airways is challenging because much of the initial human literature did not distinguish between different asthmatic phenotypes and endotypes and most were small cohorts (6). Underscoring important differences between asthma endotypes are studies showing people with neutrophilic asthma are more likely to have potentially pathogenic organisms (particularly Proteobacteria) represented in their microbiota and reduced bacterial diversity compared with people with eosinophilic asthma (59). In a study of people with severe chronic IgE-mediated asthma, the bacterial composition of the bronchial mucosa and bronchial aspirates (i.e., BALF) demonstrated a predominance of Bacteroidetes and Firmicutes with smaller contributions from Proteobacteria, Actinobacteria, and Fusobacteria (60). While cats and humans have differences in mucosal microbial communities in health and disease making direct comparisons of specific microbes problematic, it is still relevant to look for deviations from the core microbiota and determine if there is increased relative abundance of potentially pathogenic organisms or depletion of beneficial commensal populations.

One of the major hurdles in studying the lower airway microbiota in humans with asthma is that especially in pediatric patients invasive sampling to collect BALF is generally not performed (61). Instead, surrogates such as throat swabs, induced sputum (coughed from the lungs, but traversing the oropharynx) and stool samples are used to characterized respiratory dysbiosis (62). Reports of microbial composition have been shown to vary depending on the sample analyzed (63), consistent with our study. In our feline model comparing the microbial community composition from OP swabs, BALF and rectal swabs in PCoA plots showed that there were unique microbial communities that differed from each other both by site and by time. Additionally, as cats transitioned from health to acute and chronic asthma, there were no significant differences in the relative abundance at the level of phylum in OP samples, whereas there were significant changes in the BALF. Thus, without a direct sample of microbial populations from the lung, interpretation of data obtained from other sites as surrogate markers for study of the airway microbiota must be done cautiously. Another limitation of this study is the lack of metagenomic data limiting our ability to comment on the functional capability of the microbes present in asthma and health.

The current study documented temporal changes in richness and microbial community composition of the respiratory and gastrointestinal tracts as cats transitioned from health to asthma. Additionally, they provided further data to support the existence of a gut-lung axis, and the relationship between these two mucosal sites. These results provide data to further support the relevance of the feline model of asthma for the study of allergic asthma in people and document that airway inflammation can precipitate dysbiosis in the airways and lead to dysbiosis in distant sites such as the gastrointestinal tract. Obtaining a deeper understanding of the microbial interactions between these two sites, as well as the influence these organisms have on the immune system could help elucidate novel therapeutic strategies both cats and people with allergic asthma. Future studies could include characterization of these microbial populations in a larger population of cats with spontaneous asthma, as they would presumptively be more representative of human allergic asthma. The addition of metabolomic and immunologic assays in both airway and gastrointestinal samples can advance the goal of providing further insight into mechanisms through which airway dysbiosis can impact distant sites *via* immunomodulation.

## Data availability statement

The datasets presented in this study can be found in online repositories. The names of the repository/repositories and accession number(s) can be found below: https://www.ncbi.nlm.nih.gov/, PRJNA833736.

## Ethics statement

The animal study was reviewed and approved by University of Missouri Animal Care and Use Committe.

## Author contributions

AV-P participated in the conception and design of the study, sample collection, DNA extraction, data analysis, interpretation, and drafted manuscript. AE participated in the conception and design of the study, interpreted sequence data, and helped to draft the manuscript. HR assisted with DNA extraction, sample collection, and study coordination. ZM assisted with data analysis and contributed to the manuscript. CR participated in development of the animal model, conception and design of the study, sample collection, data analysis, and helped to draft the manuscript. All authors read and approved the final manuscript.

## Funding

This research was partially funded by a grant from the Veterinary Comparative Respiratory Society.

## Conflict of interest

The authors declare that the research was conducted in the absence of any commercial or financial relationships that could be construed as a potential conflict of interest.

## Publisher's note

All claims expressed in this article are solely those of the authors and do not necessarily represent those of their affiliated organizations, or those of the publisher, the editors and the reviewers. Any product that may be evaluated in this article, or claim that may be made by its manufacturer, is not guaranteed or endorsed by the publisher.

## Supplementary material

Supplementary Figure 1 **(A)** Rarefaction curves across each sample site. The dotted line indicates rarefaction depth of 1488 features. **(B)** Good's estimated coverage across each sample site. Bars indicate mean ± SD.

Supplementary Figure 2 Relative abundance of taxa present at >1% in bronchoalveolar lavage fluid (BALF) collected as 8 cats transitioned from health to acute and chronic asthma, annotated to the taxonomic level of phylum (top) and family (bottom).
