# Supplementary figures and images for: Temporal changes of the respiratory microbiota as cats transition from health to experimental acute and chronic allergic asthma
Source: Front Vet Sci. 2022 Aug 25;9:983375. doi: 10.3389/fvets.2022.983375 (PMC9453837; doi:10.3389/fvets.2022.983375)

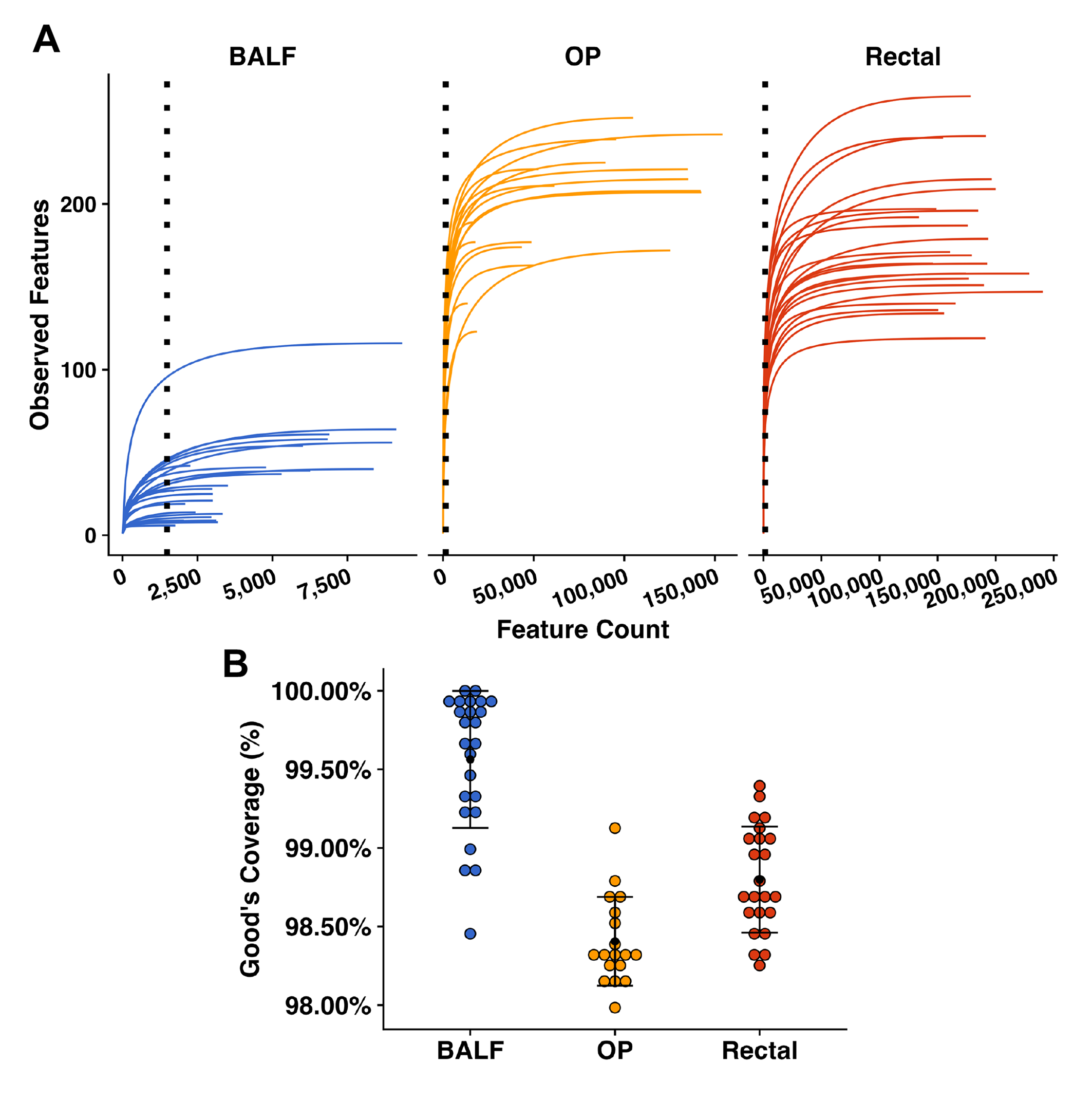

Supplement: Supplementary Figure 1 — (A) Rarefaction curves across each sample site. The dotted line indicates rarefaction depth of 1488 features. (B) Good's estimated coverage across each sample site. Bars indicate mean ± SD. [file Image_1.TIF]

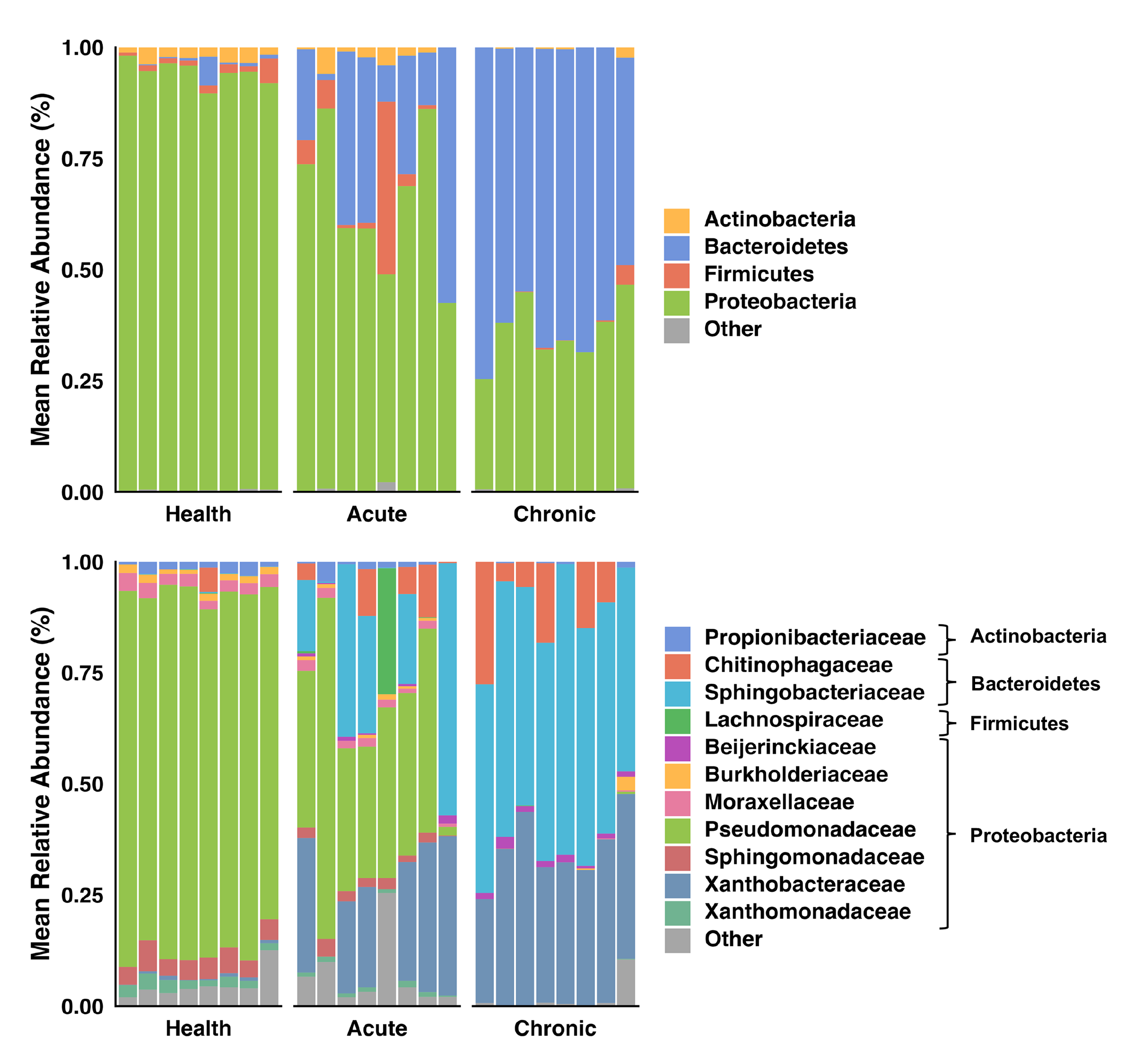

Supplement: Supplementary Figure 2 — Relative abundance of taxa present at >1% in bronchoalveolar lavage fluid (BALF) collected as 8 cats transitioned from health to acute and chronic asthma, annotated to the taxonomic level of phylum (top) and family (bottom). [file Image_2.TIF]
